# Iatrogenic Migration of an Impacted Pharyngeal Foreign Body of the Hypopharynx to the Prevertebral Space

**DOI:** 10.1155/2011/274102

**Published:** 2011-12-07

**Authors:** Jiannis Hajiioannou, Panagiotis Kousoulis, Vassiliki Florou, Eleni Stavrianou

**Affiliations:** Department of Otorhinolaryngology, General Hospital of Nikaia-Piraeus, Mantouvalou Street, Nikaia, 18454 Piraeus, Greece

## Abstract

Impaction of foreign bodies in the upper aerodigestive tract is commonly encountered in ENT practice. The present paper describes an iatrogenic complication with migration of an impacted foreign body (chicken bone) of the hypopharynx into the prevertebral space, after unsuccessful attempt of endoscopic removal. The foreign body was visualized with cervical CT scan lying extraluminally between the major vessels of the neck. An open surgical procedure with neck exploration was necessary for the definite treatment.

## 1. Introduction

Impaction of foreign bodies in the upper aerodigestive tract is a serious pathologic condition in ENT practice and is particularly common in children, prisoners, and psychiatric patients [1]. The potentially fatal complications include mediastinitis, tracheoesophageal fistula, and retropharyngeal abscess, which can lead to septicemia and shock and require immediate treatment. Although the removal of the foreign bodies through endoscopic procedures is the preferred method of treatment, this is not always possible or uncomplicated. We present a case of iatrogenic complication with migration of an impacted foreign body into the prevertebral space, after an unsuccessful attempt of endoscopic removal.

## 2. Case Report

A 44-year-old female patient visited the emergency department of a local municipal hospital with severe symptoms of dysphagia and odynophagia after ingestion of chicken bone. She underwent flexible esophagoscopy for diagnostic and therapeutic reasons, during which the foreign body was initially visualized in the hypopharynx, but iatrogenically impacted in the posterior pharyngeal wall, in a position where it was no longer possible to be removed through the esophagoscope. 

**Figure 1:**
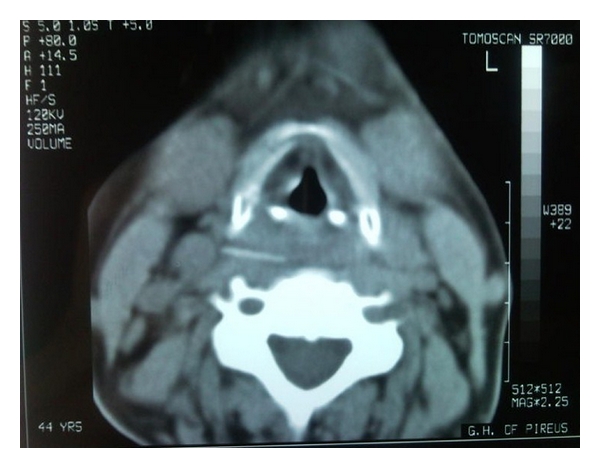
CT Imaging revealed the foreign body impacted in the prevertebral space.

The staff in the municipal hospital lacked the expertise of performing rigid esophagoscopy or open neck exploration and the patient was referred to our hospital at the same day for further treatment. On admission, the patient complained of dysphagia and odynophagia, without signs of respiratory distress. However, she had an elevated temperature of 39.5°C with a rise in white blood cell count indicating an inflammatory process. She was heavy smoker and her medical history was significant only for episodes of acute bronchitis, for which she occasionally received oral antibiotics and bronchodilators. On clinical examination pharyngoscopy, indirect laryngoscopy and direct laryngoscopy with the flexible laryngoscope were normal, although difficult to perform, due to the patients symptoms. Plain cervical and thoracic X-rays did not reveal signs of the foreign body or retropharyngeal and mediastinal emphysema. The foreign body was finally visualized in the cervical CT scan impacted extraluminally in the retropharyngeal space between the right common carotid artery and jugular vein, in contact with the anterior surface of the C5 vertebral body. No signs of air in the surrounding tissues were present (Figure 1).

**Figure 2:**
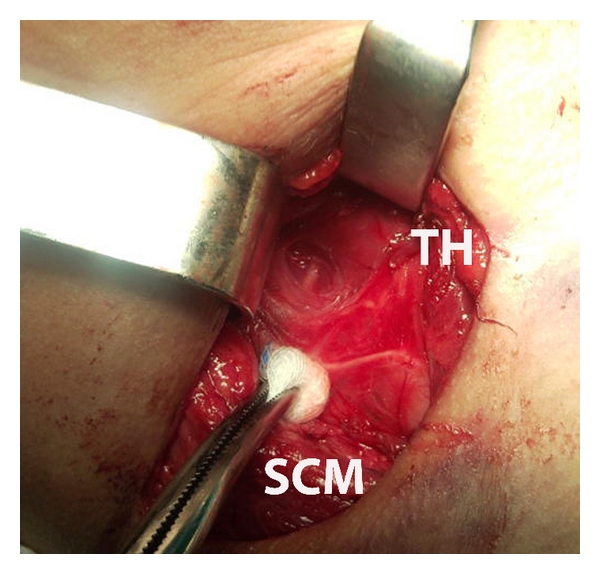
Intraoperative view with the foreign body in the center of the surgical field. The thyroid gland (TH) and the sternocleidomastoid muscle (SCM) have been retracted. The recurrent laryngeal nerve crosses the surgical field.

**Figure 3:**
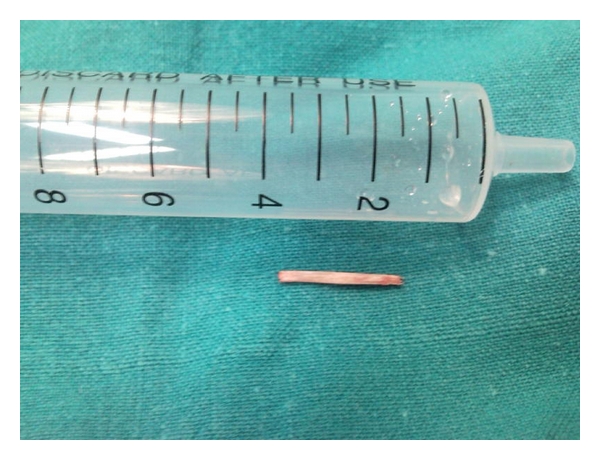
The impacted chicken bone after the surgical removal.

In view of the clinical signs and symptoms and in order to preclude a deterioration of the patients' condition and a possible abscess formation a prompt removal of the bone under general anaesthesia was decided. A rigid esophagoscopy was not performed at this stage as it was considered unnecessary due to the extraluminal position of the foreign body and carried more risk of enlarging the perforation. Through a right cervical incision, retraction of the sternocleidomastoid muscle, and the carotid sheath, the bone was palpated in the prevertebral space where it had further migrated and was removed (Figures 2 and 3). No pharyngeal wall laceration was noticed. The soft tissues were irrigated, a vacuum surgical drain was placed, and the incision was closed in layers with interrupted sutures. A nasogastric tube was placed and remained for 24 hours. Broad-spectrum antibiotics were administered postoperatively intravenously for 72 hours until fever resolved. The drain was removed in the 2nd postoperative day and the patient was discharged home.

## 3. Discussion

Impaction of foreign bodies in the upper food and airway passage is usual in otolaryngological practice. Commonly found objects include fish bones, chicken bones, pieces of glass, dental prostheses, coins, and needles [2–6]. The morbidity of the impacted foreign objects relates to their size, shape, and site of impaction. Although the majority of the small and round ones pass along the gastrointestinal tract spontaneously, the sharper and larger objects can lead to serious complications. Perforation of the pharyngeal or esophageal wall is possible, and migration of the foreign body in the adjacent tissues can be facilitated by swallowing, coughing, and esophageal peristalsis [7], as well as by the weakening of the pharyngeal wall due to the local inflammation [8]. Complications from perforation of the hypopharynx and upper esophagus include retropharyngeal abscess [2, 5], mediastinitis, and even rare instances of esophagoarterial fistulas [9, 10], penetration of the common carotid [11] and facial artery [12], thyroid abscess [13], and epidural abscess [14]. Most authors agree that delayed diagnosis and retrieval is associated with an increased complication rate [15–17]. 

Pain in the form of odynophagia is the most constant symptom, although retrosternal pain and painful cervical contracture can also be observed [15, 17]. Foul smelling expectoration and muffled voice are symptoms of abscess formation and subsequent clinical deterioration and are common in patients seeking late medical attention [12, 17]. Drooling, dysphagia, dysphonia, and dyspnea due to airway obstruction can also be observed, depending on the site of impaction [5, 6]. Increased body temperature, shiver, cervical crepitance, and cervical swelling are signs of complications of the foreign body and should alert the clinician for urgent treatment [12]. In the present case, signs of bacterial complication (leucocytosis and fever) were evident, although the patient presented to our hospital within a few hours from the impaction. 

Diagnosis is based on history and symptoms, but visualization of the foreign body and evaluation of the possible complications often require the use of a variety of diagnostic procedures. Indirect laryngoscopy, soft tissue lateral neck X-rays, routine chest X-rays, oesophagography with gastrografin, barium swallow, CT scan, and endoscopy through flexible and rigid endoscopes can be used, each one having advantages and disadvantages [17, 18]. Since the majority of foreign bodies are impacted in the suprahyoid region [19], they are usually detected by indirect laryngoscopy alone and can be removed with local anesthesia in the outpatient setting. 

Plain radiography has the benefit of evaluating the deeper soft tissues and revealing potential complications. The physician should look for abnormal calcifications in the cervical region, cervical or mediastinal emphysema, widening of the prevertebral soft tissue space, and presence of soft tissue swelling in the region of the base of the tongue [17, 20]. 

On the other hand, CT scanning of the neck is considered the most accurate imaging modality for diagnosing the presence of any foreign body impaction and superior to the plain radiogram [21, 22]. Studies have shown that sensitivity and specificity of plain X-ray for the detection of fish bones range between 23.5% and 54.8% and 86.3 and 100%, respectively [19, 23], whereas non-contrast-CT has a sensitivity of over 90% and specificity of 100% [23, 24]. Some authors recommend its use in complicated cases [8], while others advocate that CT should be performed in all cases of suspected foreign body which cannot be visualized through laryngeal mirror or laryngeal fiberscope [23]. The high radiation dose compared to plain radiography is a serious disadvantage of the CT scan [18], and sound clinical judgment is required in order to be used in selected patients, under the “do no harm” principle. In the present case, the CT scan was the diagnostic method of choice, since the foreign body was impacted extraluminally in the posterior pharyngeal tissues. 

Endoscopy has the advantage of direct visualization of the mucosal layer under magnification to assess for signs of trauma. A flexible endoscope with a lumen for insertion of grasping instruments is required in order to be used therapeutically, as well as diagnostically. In a recent study [16], the success rate of flexible endoscopy for foreign body extraction was 98%. The authors reported complications such as lacerations (6.7%) and perforation (1%) of the esophagus occurring during the endoscopic procedure. In our case, the foreign body was impacted further into the retropharyngeal space during the endoscopic procedure, in a position where it was no longer visible through the endoscope. The authors are unaware of a report of a similar complication in the published literature. 

Clinical management of impacted foreign bodies in the hypopharynx and upper esophagus focuses on preserving the airway, removing the foreign body, and treating any complications that may arise. An open surgical procedure with neck exploration is mandated when endoscopic removal fails, as in the present case, and when infectious complications, such as abscess formation and mediastinitis, have developed. After removal of the foreign body, primary closure of a perforation can be attempted and the neck should be thoroughly irrigated and drained. Postoperative antibiotic therapy is necessary in order to prevent further complications. 

Impacted foreign bodies in the upper respiratory tract and upper esophagus are common in otolaryngology practice. Endoscopic removal is the treatment of choice of the symptomatic cases, although not always possible or without complications. The open surgical procedure with careful neck exploration is an alternative approach when other treatments fail or when complications arise. A prompt diagnosis and early therapeutic intervention is required in order to minimize morbidity and mortality from the impacted foreign bodies.
